# Effect of polishing process on torque loss ratio and microgap of selective laser melting abutment: an in vitro study

**DOI:** 10.1186/s12903-024-04829-y

**Published:** 2024-09-09

**Authors:** Peixing Zhong, Limei Deng, Sheng Xu, Yong Cao

**Affiliations:** 1 Department of Prosthetics, Guangxi Medical University College of Stomatology, 6 Shuangyong Road, Nanning, 530021 Guangxi PR China; 2 Department of Oral and Maxillofacial Surgery, The People’s Hospital of Guangxi Zhuang Autonomous Region, 6 Taoyuan Road, Nanning, 530021 Guangxi PR China

**Keywords:** Implant-abutment interface, Microgap, SLM abutment, Polishing process, Surface roughness, Torque loss ratio

## Abstract

**Background:**

The purpose of this in vitro study was to investigate the effect of polishing post-treatment process on the torque loss ratio and microgap of Selective Laser Melting (SLM) abutments before and after mechanical cycling test through improving the surface roughness of the implant-abutment interface.

**Materials and methods:**

Forty SLM abutments were fabricated, with 20 underwent minor back-cutting, designated as polishing, in the implant-abutment interface. The abutments were divided into three groups: SLM abutments (group A), original abutments (group B), and polished SLM abutments (group C), each containing 20 abutments. Surface roughness was evaluated using a laser microscope. Implant-abutment specimens were subjected to mechanical cycling test, and disassembly torque values were measured before and after. Scanning electron microscope (SEM) was used to measure microgap after longitudinal sectioning of specimens. Correlation between surface roughness, torque loss ratio, and microgap were evaluated. LSD’s test and Tamhane’s T2 comparison were used to analyze the data (α = 0.05).

**Results:**

The Sz value of polished SLM abutments (6.86 ± 0.64 μm) demonstrated a significant reduction compared to SLM abutments (26.52 ± 7.12 μm). The torque loss ratio of polished SLM abutments (24.16%) was significantly lower than SLM abutments (58.26%), while no statistically significant difference that original abutments (18.23%). The implant-abutment microgap of polished SLM abutments (2.38 ± 1.39 μm) was significantly lower than SLM abutments (8.69 ± 5.30 μm), and this difference was not statistically significant with original abutments (1.87 ± 0.81 μm). A significant positive correlation was identified between Sz values and the ratio of torque loss after cycling test (*r* = 0.903, *P* < 0.01), as well as Sz values and the microgap for all specimens in SLM abutments and polished SLM abutments (*r* = 0.800, *P* < 0.01).

**Conclusion:**

The findings of this study indicated that the polishing step of minor back-cutting can lead to a notable improvement in the roughness of SLM abutments interface, which subsequently optimized the implant-abutment fit. It can be seen that the application of minor back-cutting method has advanced the clinical use of SLM abutments.

## Background

Implant restoration stands as the preferred approach for addressing missing teeth. Conventional abutments from implant manufacturers, alongside castable and cuttable abutments, currently dominate clinical practice. Standard abutments, with a fixed form, may lack the necessary personalization for precise implant sites [1]. Castable and cuttable abutments, on the other hand, are employed when deviations in implantation sites necessitate modification for optimal restoration [2]. However, their clinical utility is hampered by high costs and material waste. Thus, a demand persists for a customized abutment with cost-effective and easily fabricated, in order to enhance the success rate of implant restorations.

SLM is the additive manufacturing technology that involves collecting data from computer-aided design/computer-aided manufacturing (CAD/CAM) to create a digital model and then molding the powdered material in a layer-by-layer stacking fashion [3]. This digital technology is widely used in the fabrication of metal parts of oral restorations, such as crowns, bridges, frameworks, implants, abutments, etc. [4, 5]. SLM and other additive techniques provide abutments with several advantages, including high productivity, low cost, excellent mechanical properties, and a high degree of customization [6–8]. However, incomplete fusion of powder particles that adhere to the component surfaces during abutment fabrication can cause a ballooning effect. This results in a large microgap between the SLM abutment and the implant, which limits the clinical application of the SLM abutment [9, 10]. Residual powder particles on the abutment’s surface increase roughness at the implant-abutment interface, impacting the precise seating of the abutment [8, 9]. The microgap, a small gap between the implant-abutment interface in two-stage implantology, ranging from 0.55 to 50 μm in different studies [11]. The diameter of common oral bacteria falls within the range of 0.8–6.0 μm [12]. Consequently, most bacteria can enter and colonize the implant-abutment interface through the microgap, potentially causing peri-implantitis and bone resorption in the neck [13]. Additionally, the microgap reflects misfit between the implant and abutment, contributing to mechanical complications such as screw loosening in a long-term oral masticatory environment [14, 15]. Markarian et al. [16] conducted a simulated oral mastication to compare the microgap between external connected implants and different abutments, they found that SLM abutments exhibited the largest microgap values. Similarly, in the internal connection system, the study by Gonzalo [7] and Mourelle et al. [17] demonstrated that the microgap value of CAD/CAM abutments was the maximum among all groups. Markarian et al. via cyclic loading experiments found that the primary factor influencing the precision of the accuracy and the wear of CAD/CAM abutments was the high surface roughness [18]. The irregularities on the surface affected the clamping force generated by the fixation screw during abutment installed onto a dental implant, thus compromising the accuracy of SLM abutment seating [8]. Thus, the study focused on decrease the surface roughness of the implant attachment surface of the SLM abutment to enhance its accuracy during seating with the implant.

Nevertheless, SLM abutments still have wide range of clinical applications due to their various advantages such as high fabrication efficiency, low cost, and a high degree of personalization. When angular abutments fail to meet clinical needs, SLM technique allows the creation of ideal angular abutments. Metal-printed parts produced by additive manufacturing can be subsequently milled through computer numerical control to achieve a smooth finish [19]. The implant-platform/framework interface for full-arch dental restoration on implants, processed using a hybrid technique that combines SLM and milling, achieves greater trueness and precision [20]. We presumed that minor back-cutting, as a polishing post-processing step, improves the accuracy of SLM abutments. In this approach, abutment blanks are generated by SLM technique, and the abutment interface is subsequently polished by minor back-cutting. This method not only preserves the customization of the abutment but also enhances the accuracy of SLM abutments to some extent during seating with the implants. However, there is a lack of reports on the implant-abutment interface accuracy of milling process to SLM abutments, necessitating further study to evaluate the fitness of such abutments and their microgap values after cyclic loading. The purpose of this study was to evaluate the feasibility of polishing as a post-processing step of SLM abutment to enhance the accuracy. The null hypothesis was that, polishing or not would be no significant difference in roughness and microgap at the implant-abutment interface of SLM abutment.

## Materials and methods

### Experimental design

The experiment was divided into the following groups (*n* = 20): group A: SLM abutments; group B: original abutments; group C: polished SLM abutments.

**Fig. 1:**
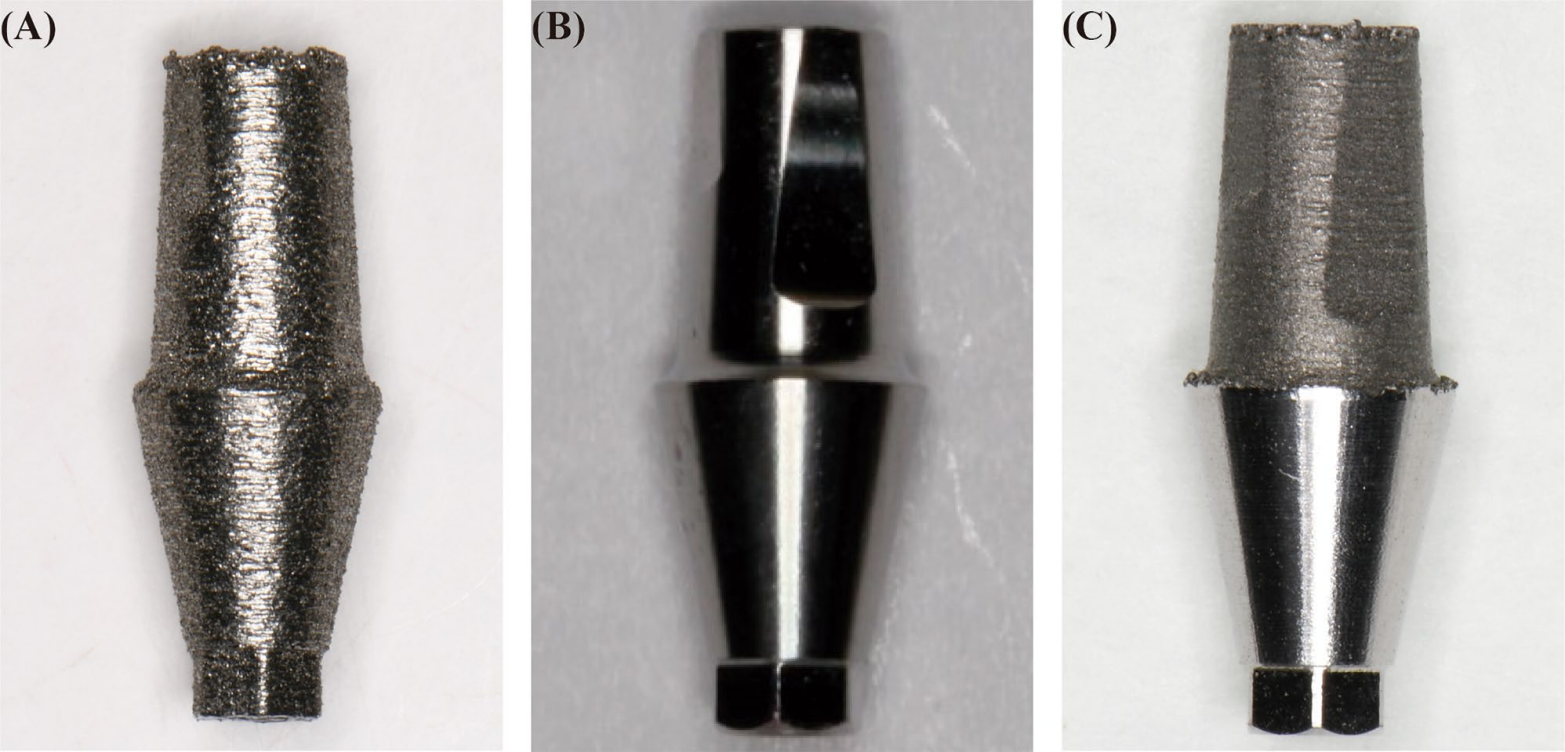
Experimental grouping of abutment. (**A**) SLM abutment, (**B**) Original abutment, (**C**) Polished SLM abutment

Obtained the standard tessellation language (STL) data of the straight abutment from the manufacturer (Guangdong Casangels Biotechnology Co., Ltd, Foshan, China) and transferred it to the three-dimensional (3D) modeling software. In the experimental group (Group C), the internal connection of the abutment was customized to enlarge 100 μm uniformly, accounting for the precision of the SLM machine and the milling machine, as well as the outcomes of the preliminary results of abutment interface roughness experiment (40.39 ± 8.56 μm). However, further research is necessary to ascertain the optimal amount. The resulting STL files were used to create 20 SLM abutments and 20 customized SLM abutments through the utilization of a laser rapid prototyping machine (PST-L100, Guangdong Pstphoton Photonics Technology Co., Ltd, Zhongshan, China) and a titanium alloy powder as raw material, employing rapid prototyping technology. These customized SLM abutments were further polishing by a minor back-cutting method at their internal connection part through a five-axis milling machine (Wieland Zenotec Select Hybrid, Ivoclar Vivadent AG, Schaan, Liechtenstein). The polishing post-treatment step served for customized SLM abutment to achieve a size identical to the original abutment. Finally, group A, group B, group C were prepared and contained 20 SLM abutments, original abutments and polished SLM abutments, respectively (Fig. 1). All abutments were considered compatible with Angels dental implants (4.3 × 10 mm) (AI0143100A, Guangdong Casangels Biotechnology Co., Ltd, Foshan, China). The universal implant has favorable biocompatibility, which features a 12° taper and is attached to the abutment via a Morse taper connection structure.

### Detection of abutment surface roughness

The maximum height (Sz) value was acquired by employing a laser microscope (VK-X1000 Series, KEYENCE, Japan) to gauge surface roughness at the implant-abutment interface of each abutment, measuring three regions that were 120-degree angle of angulation apart. Subsequently, computer-generated surface topography and 3D contours were produced to assess alterations in surface roughness at the implant-abutment interface before and after the polishing process.

### Torque loss ratio experiment before and after mechanical cycling

**Fig. 2:**
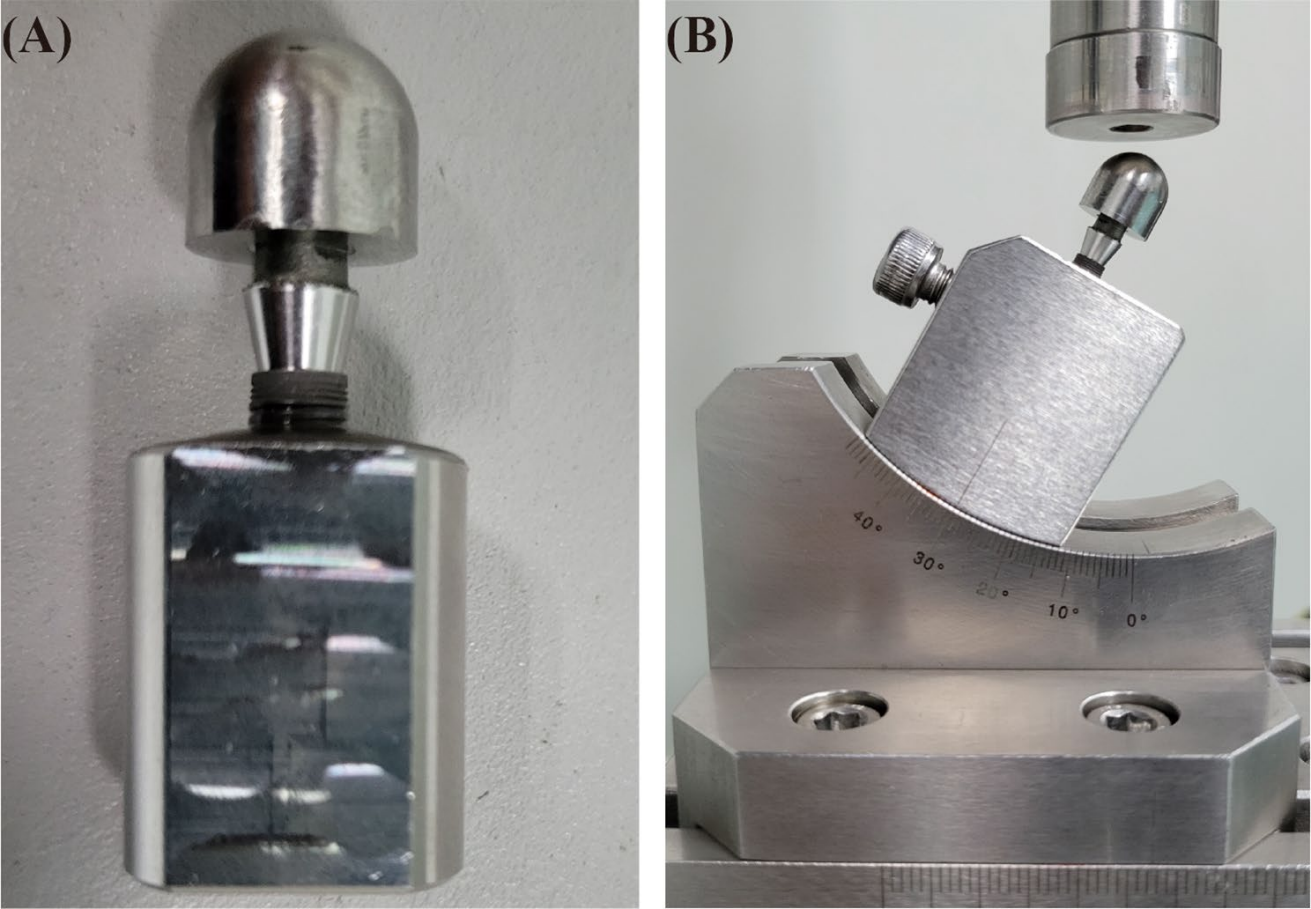
Determination of cyclic loading direction. The implant-abutment specimens positioned by customized made metal fixture (**A**) and set at 30-degree angle to the loading direction (**B**)

Sixty implants were selected and randomly fixed with an abutment in each group to form the implant-abutment specimens. To comply with the recommended torque specified by the manufacturer (RTM), each assembly was first secured to the clamp and then the abutment screw was torqued to 30 N·cm. Ten specimens from each group were tightened again at the manufacturer’s recommended torque after 10 min, compensating for preload loss due to the settling effect. The Removal Torque Value 1 (RTV1) necessary to loosen the assembly was recorded with an electronic torque meter (SDE-05BN, WIZTANK, Taiwan). Subsequently, the specimens were retightened at the manufacturer’s recommended torque and then positioned in custom-made metal fixtures (Fig. 2A) (TC4, Guangdong Casangels Biotechnology Co., Ltd, Foshan, China). To simulate masticatory forces, the abutment-implant assembly was subjected to 10^6^ mechanical cycles (E3000, Instron, High Wycombe, UK) at a frequency of 2 Hz under 200 N and 30-degree angle. (Fig. 2B). The Removal Torque Value 2 (RTV2) required for unscrewing after mechanical cycling was then recorded using an electronic torque meter. The torque loss ratio for all specimens was subsequently calculated both before and after mechanical cycling using the following equation respectively:

$$\:\text{T}\text{o}\text{r}\text{q}\text{u}\text{e}\:\text{l}\text{o}\text{s}\text{s}\:\text{r}\text{a}\text{t}\text{i}\text{o}\:\text{b}\text{e}\text{f}\text{o}\text{r}\text{e}\:\text{l}\text{o}\text{a}\text{d}\text{i}\text{n}\text{g}=\frac{\text{R}\text{T}\text{M}-\text{R}\text{T}\text{V}1}{\text{R}\text{T}\text{M}}\times\:100\%$$

$$\:\text{T}\text{o}\text{r}\text{q}\text{u}\text{e}\:\text{l}\text{o}\text{s}\text{s}\:\text{r}\text{a}\text{t}\text{i}\text{o}\:\text{a}\text{f}\text{t}\text{e}\text{r}\:\text{l}\text{o}\text{a}\text{d}\text{i}\text{n}\text{g}=\frac{\text{R}\text{T}\text{M}-\text{R}\text{T}\text{V}2}{\text{R}\text{T}\text{M}}\times\:100\text{\%}\:\text{o}\text{f}\:\text{t}\text{h}\text{e}\:\text{t}\text{o}\text{t}\text{a}\text{l}$$

### Measurement of microgap after mechanical cycling

The rest of ten specimens from each group also underwent mechanical cycling test, as previously described. Then these specimens were embedded with metallographic inlay (XBH-30, Shanghai Xinbiao Testing Instrument Manufacturing Co., Ltd, Shanghai, China). After curing, longitudinal slices were obtained from the largest diameter of the implant-abutment using a precision cutting machine (Q15001, Guangdong goral Industrial Co., Ltd, Dongguan, China). The surface of each section was then polished with a metallographic specimen grinding and polishing machine (MP-2B, Guangdong goral Industrial Co., Ltd, Dongguan, China). Debris was cleaned using ultrasound and a high-pressure air gun. Subsequently, six points at implant-abutment interface were selected for each specimen, including buccal external point, middle point, internal point, and lingual external point, middle point, internal point, and then their microgaps were observed using SEM (GeminiSEM 500, Zeiss, Germany). The images were imported into an image measuring pixel.

counting software (Image J, National Institutes of Health, MA, USA) for microgap measurement. To avoid the potential for researcher bias, each point was measured five times by the same individual. The means and standard deviations of the microgap were calculated for each point and compared to the overall means of the microgap values for each group.

### Statistical analysis

The data were subjected to analysis using SPSS software (IBM SPSS Statistics, v25, USA) and the GraphPad Prism software (GraphPad Software Inc., v9, USA). The mean and standard deviations were calculated for each group. The Shapiro-Wilk test was used to evaluate the distribution of the data. A one-way ANOVA analysis was conducted to examine the homogeneity of variance. The LSD test was selected for post hoc multiple comparisons when variance was aligned, otherwise the Tamhane’s T2 method was utilized. Pearson correlation test was employed to evaluate the possible correlation between Sz and torque loss ratio, as well as and microgap. All statistical tests were bilateral, with the level of significance was α = 0.05.

## Results

**Fig. 3:**
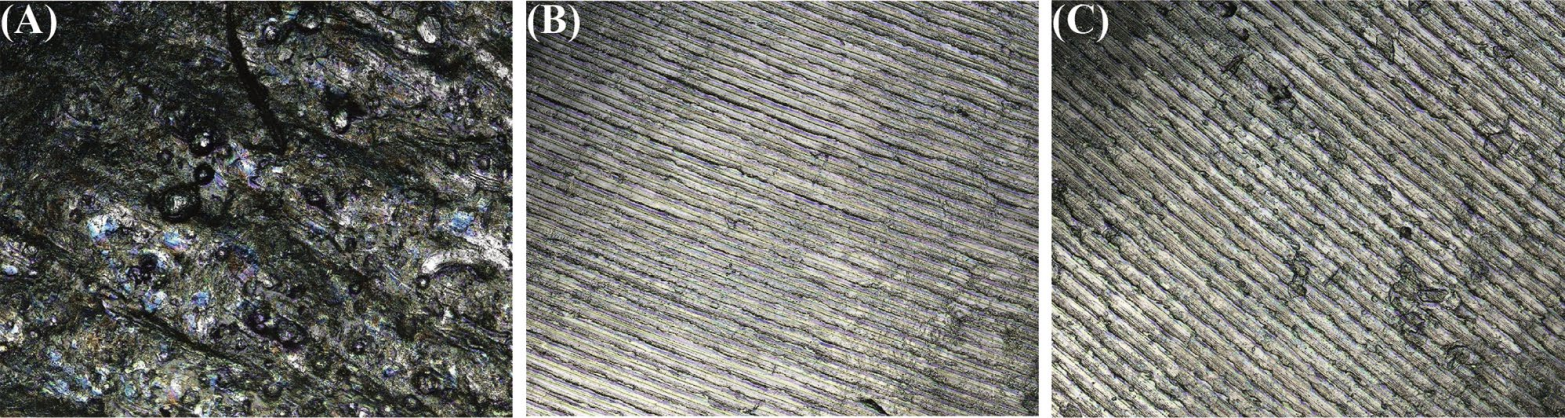
Surface topography of abutment. (**A**) SLM abutment, (**B**) Original abutment, (**C**) Polished SLM abutment

**Fig. 4:**
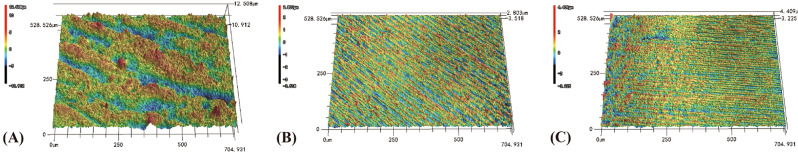
3D contour maps of abutment surface roughness. (**A**) SLM abutment, (**B**) Original abutment, (**C**) Polished SLM abutment

**Fig. 5:**
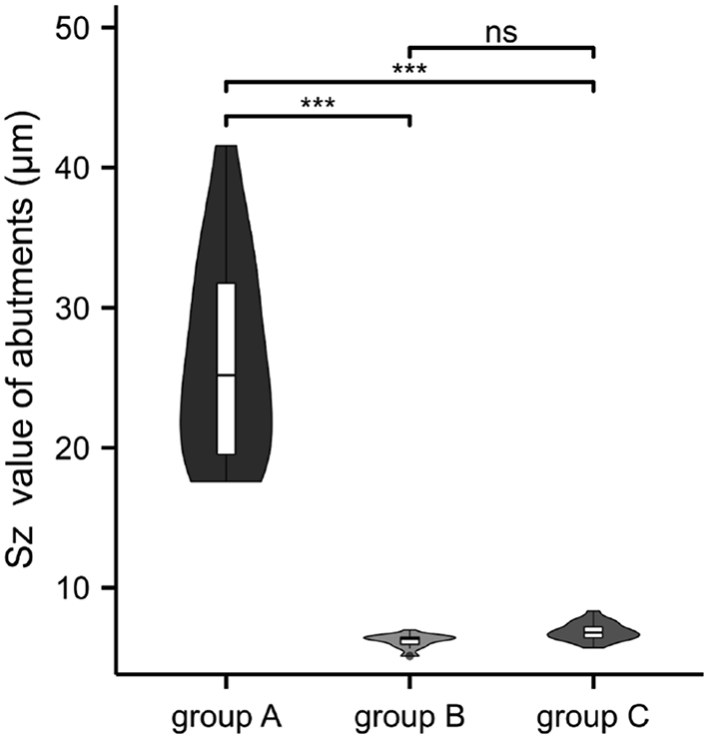
Surface roughness (Sz value) of abutments, experimental data is expressed as mean ± standard deviation, *n* = 20, ns: no statistical significance, ***:*P*<0.001

To achieve a smoother implant-abutment interface, the SLM abutment underwent a polishing process. As anticipated, the surface topography and 3D contour maps showed a marked reduction in the abutment’s surface roughness after polishing (Figs. 3 and 4). The Sz values of each group were as follows: 26.52 ± 7.12 μm, 6.19 ± 0.48 μm, 6.86 ± 0.64 μm. The violin plot revealed significant differences between groups A and B, as well as groups A and C (P *<* 0.05); however, no statistically significant differences were observed between groups B and C (*P* > 0.05) (Fig. 5). This illustrated that the surface roughness of SLM abutment after polishing process was significantly reduced, approaching the level of the original abutment.

**Fig. 6:**
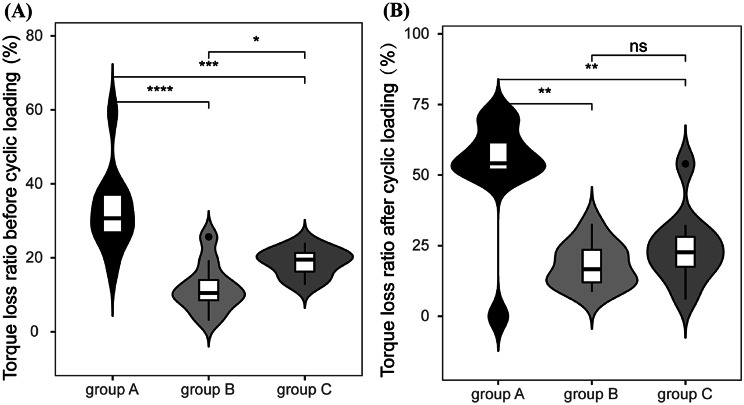
Torque loss ratio (%) of each group before and after mechanical cycling, experimental data is expressed as mean ± standard deviation, *n* = 10, ns: no statistical significance, *: *P*<0.05, **:*P* < 0.01, ***:*P* < 0.001. (**A**) Torque loss ratio before mechanical cycling, (**B**) Torque loss ratio after mechanical cycling

The disassembly torque values and torque loss ratios before and after cyclic loading test for groups A, B, and C were presented in Table 1. The statistically difference between each group was visually exhibited in the violin plot (Fig. 6), supporting the conclusion that the polishing process strengthen the accuracy of SLM abutment connection.

**Table 1:** Removal torque value and torque loss ratio of abutments before and after mechanical cycling

	*n*	RTM (*N*·cm)	RTV1(*N*·cm)		Torque loss1 (%)	RTV2(*N*·cm)		Torque loss2 (%)
			Mean	SD		Mean	SD	
Group A	10	30	20.12	3.36	32.93	12.44	2.31	58.26
Group B	10	30	26.47	2.02	11.89	24.54	2.30	18.23
Group C	10	30	24.38	1.09	18.73	22.87	3.93	24.16

RTM: recommended torque specified by the manufacturer

RTV1: removal torque value necessary to loosen the assembly before mechanical cycling

Torque loss1: torque loss ratio before mechanical cycling

RTV2: removal torque value necessary to loosen the assembly after mechanical cycling

Torque loss2: torque loss ratio after mechanical cycling

**Fig. 7:**
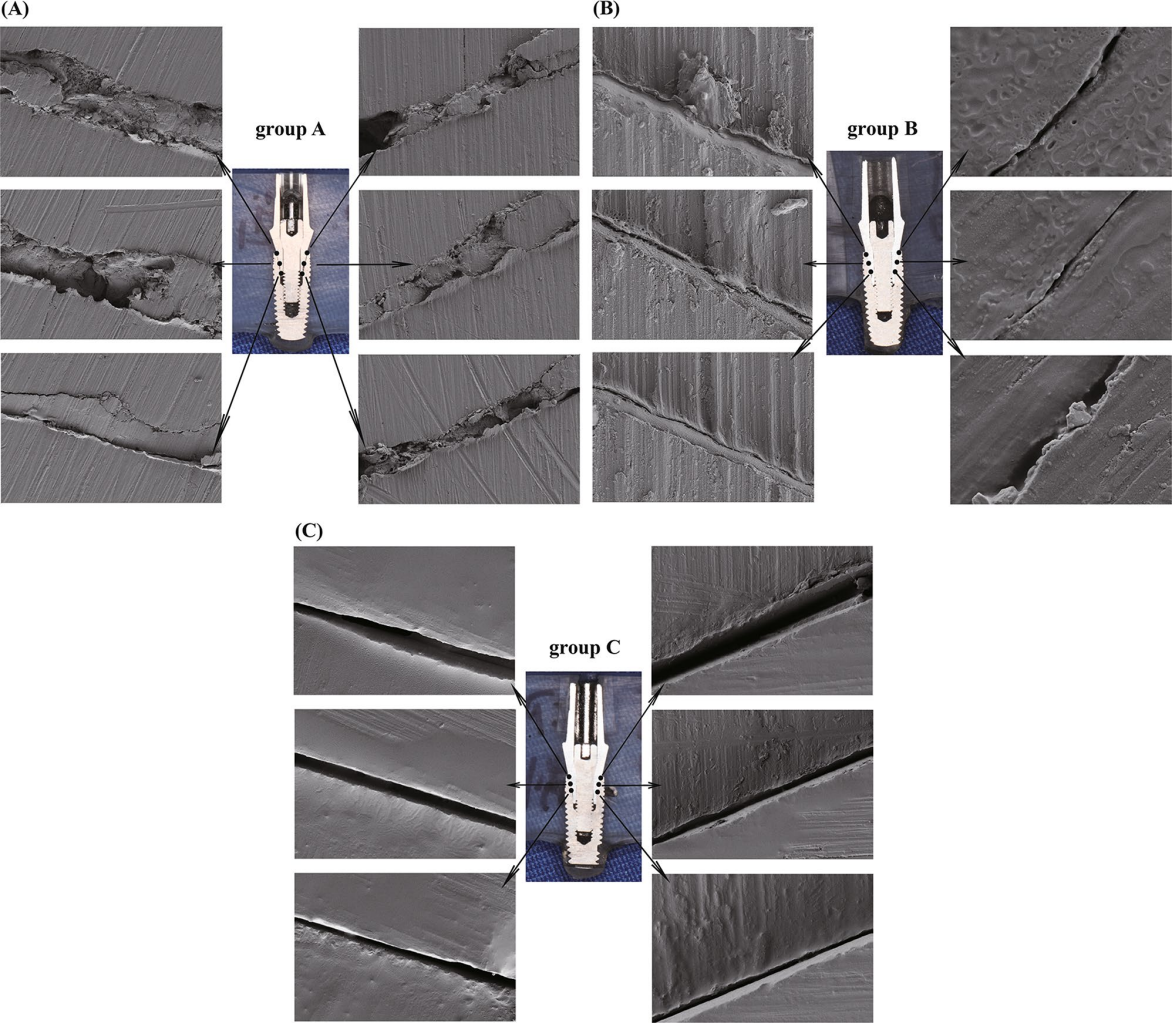
Scanning electron micrograph at implant-abutment interface of each group section. (**A**) group A: SLM abutment, original magnification×1000. (**B**) group B: Original abutment, original magnification×8000. (**C**) group C: Polished SLM abutment, original magnification×4000

**Fig. 8:**
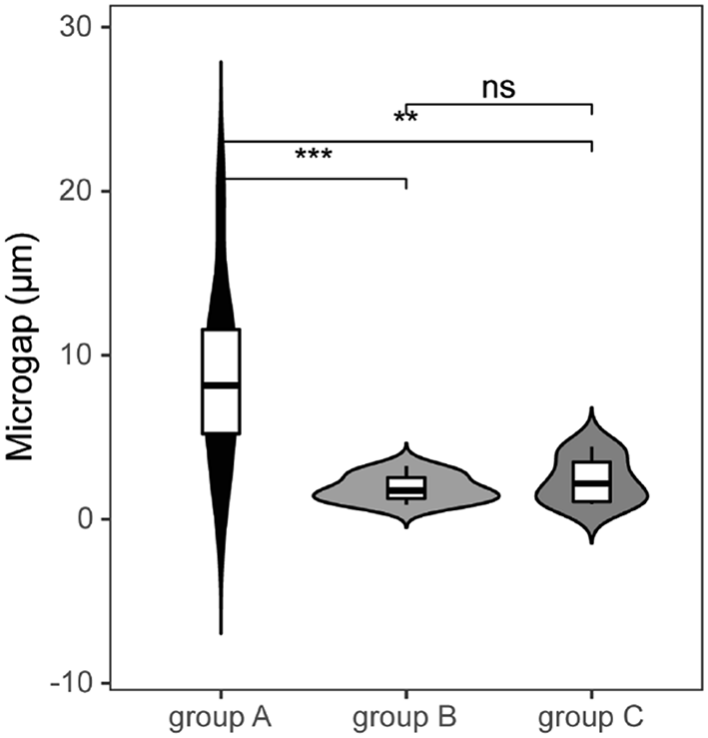
Microgap (µm) of each group, the experimental data is expressed as mean ± standard deviation, *n* = 10, ns: no statistical significance, *:*P* < 0.05, **:*P* < 0.01, ***:*P* < 0.001

After the loading test, the implant abutment specimens were cut longitudinally along the maximum diameter. Then SEM was employed to observe the microgap at each selected point within the specimen at the implant abutment interface of each group. A representative image of each point was selected for representation in Fig. 7. Specifically, the microgap of each group was 8.69 ± 5.30 μm, 1.87 ± 0.81 μm, 2.38 ± 1.39 μm, respectively. The statistically significant differences were obtained between groups A and B, and between groups A and C (P *<* 0.05); however, this difference was not statistically significant between groups B and C (*P* > 0.05) (Fig. 8).

**Fig. 9:**
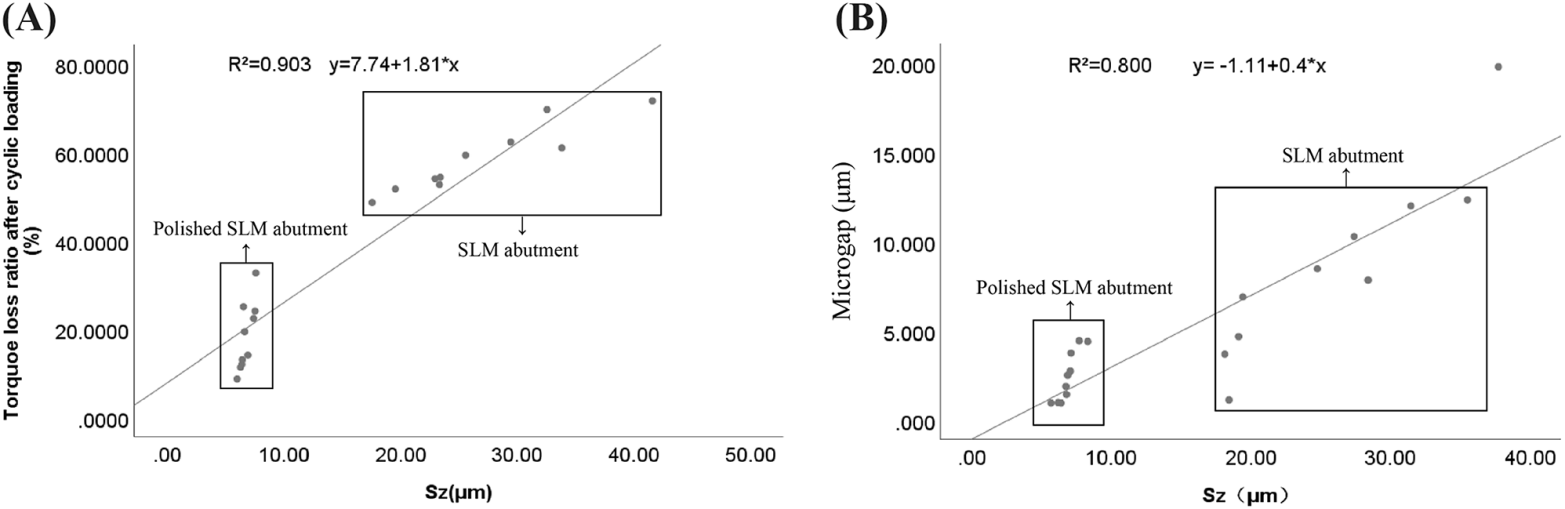
Scatter diagram between surface roughness and torque loss ratio after mechanical cycling, and between surface roughness and microgap of all SLM abutment. (**A**) surface roughness and torque loss ratio after mechanical cycling, (**B**) surface roughness and microgap

Pearson’s correlation coefficients between Sz values and the ratio of torque loss after cyclic loading, and microgap for groups A and C was 0.903 (P *<* 0.01) and 0.800 (P *<* 0.01), respectively (Fig. 9). Consequently, it is evident that there exists a significant and strong positive correlation between surface roughness and the ratio of torque loss after cyclic loading in SLM abutments, as well as between surface roughness and microgap.

## Discussion

SLM technology was able to reproduce the customized implant designs with high strength and sufficient dimensional accuracy [21]. This study adopted a composite abutment fabrication method. A special design for the group of polished SLM abutments was conducted, which enlarger 100 μm uniformly at the internal connection than the original abutments. Then a milling machine was employed to cut the additional part to match the original abutment size. This process actually carried out a minor back-cutting of the blank, which we referred to as polishing.

In this study, the SLM abutment, before polishing process, exhibited a notable high surface roughness with a mean Sz value of 26.52 μm. Following the polishing process, the mean Sz value experienced a significant reduction to 6.86 μm. This compelling difference led to the conclusion that the polishing process substantially improved the surface roughness of the SLM abutment.

The evaluation of a surface texture involves various measurement method, these instruments are based on tactile or optical methods [22]. However, one disadvantage of stylus instruments is that the stylus may damage the specimen surface [23]. To avoid surface wear and tear caused by traditional stylus-type roughness measuring instruments during contact measurements [24], particularly when the stylus traverses the SLM abutment’s surface, this study employed laser microscopy as a non-contact measurement method. And chosen surface roughness, specifically the Sz value, as measurement parameter due to the curved surface structure of the abutment connection area, instead of line roughness.

The torque loss ratio serves as a critical indicator of the fit between the implant and the abutment, offering insights into the stability of the screw connection. The screw-in torque force during abutment screwing represents the preloading force, with a higher loss indicating a poorer fit of the abutment to the implant and, consequently, reduced stability of the screw connection [25].

Before the polishing process, the torque loss ratio for the SLM abutment was 32.93%, a value that decreased to 18.73% after polishing. This reduction was not statistically significantly different from the 11.89% observed for the original abutment, suggesting a substantial improvement in the accuracy of the SLM abutment for implants after polishing process. Simulating early implantation complications, the study subjected specimens to 10^6^ cycles, approximating the reported yearly chewing frequency of per person [26, 27]. Early implant complications can manifest after a year of prosthetic function [28].

SLM abutments, characterized by high surface roughness, necessitated overcoming irregularities abutment surface during abutment screwing to reach the seating position. This results in a higher preloading force loss, which led to a mean torque loss ratio of 58.26% after cycling test. Intriguingly, the specimen with the highest torque loss ratio recorded 71.67%, which corresponds to the largest Sz value among all SLM abutments (37.741 μm). Sz, which represents an extreme value of surface roughness, can significantly affect abutment seating conditions. The study’s use of Sz to characterize roughness provides a comprehensive exploration of the relationship between abutment roughness and the accuracy.

Following polishing, the mean Sz value of the SLM abutment decreased to 6.86 μm, resulting in a torque loss ratio after cyclic loading of 24.16%. This value was significantly lower than that of the unpolished SLM abutment. Pearson correlation analysis revealed a significant and strong positive correlation between Sz and the torque loss ratio after cycling test. This finding aligns with Khraisat et al. [29], suggesting that surface roughness contributes to preloading force loss, thereby reducing connection stability.

In a similar research, Alonso-Pérez et al. [30] found that the torque loss ratio after 2 × 10^6^ cycling loading was 13.1% for the Straumann original abutment as well as 24.6% and 34.1% for the two non-original cutting abutments, aligning closely with the findings of the present experiment. Additionally, Sammour et al. [31] investigated the torque loss ratio before and after 10^5^ cyclic loading of the Morse taper internal joint implant system, resulting in a torque loss ratio after cycling of 11.47%. The lower torque loss ratio might be attributed to the experimental loading of only 10^5^ cycles, and it is noted that the torque loss ratio tends to increase with a higher cycle number. Furthermore, the magnitude of the torque loss ratio is influenced by various factors, including different implant diameters [32], connection styles [31], angles of the abutment [33], as well as different attachment methods. This diversity in influencing factors accounts for the variations in torque loss ratios reported across different studies.

Various methods have been employed in the literature for measuring the implant-abutment microgap, including direct observation by SEM [7, 17, 34, 35], micro-CT scanning [36–38] and the profile incision measurement method [30, 39]. SEM method involves fixing the implant-abutment specimen and capturing images of the microgap width at an angle perpendicular to the microgap. However, it falls short for internally connected implant systems as it cannot intuitively observe and measure the implant-abutment internal gap. Additionally, microgap values was surpass the general resolution and accuracy limits of micro-CT. Therefore, in this study, the profile incision measurement method was employed. This involved longitudinally cutting the implant-abutment specimen to obtain a profile, followed by microgap measurement through SEM. This approach was chosen for its suitability in visualizing and measuring the microgap in internally connected implant systems, addressing the limitations of other techniques in this specific context.

In this study, the average microgap measurement for the SLM abutment amounted to 8.69 μm. Following the polishing process, this metric experienced a notable reduction, settling at 2.38 μm. Thus, the null hypothesis of this study was rejected. This study draws inspiration from the work of Fernandez et al. [6], which previously established a positive correlation between surface roughness and microgap in abutments designed for externally connected implant systems. Seeking to extrapolate this correlation to internally connected implant systems, the variable Sz was employed in our study to quantify roughness. Consequently, a correlation analysis was conducted between surface roughness and microgap. The outcomes revealed a substantial and statistically significant positive correlation, as denoted by a Pearson correlation coefficient of 0.800 (*P* < 0.01). This robustly signifies the presence of a noteworthy positive association between surface roughness and microgap within the context of internally connected implant systems.

In a comparable investigation detailed by Mourelle et al. [17], the mean microgap was scrutinized across four distinct abutments and implants after 3 × 10^5^ fatigue cycles. Notably, Mourelle et al. employed a direct SEM observation method, examining buccal, lingual, proximal-medial, and distal-medial orientations at different angles, differing from the approach adopted in our present experiments. Duraisamy et al. reported that, featuring ADIN implants, disclosed that the average microgap at the Morse taper junction for the original abutment was 1.61 μm, while the milling non-original abutment registered an average microgap of 2.74 μm [40]. Sui et al. documented an average microgap at the Mohs taper junction between zirconia abutment based of the original abutment scanning data, and Osstem implant, as 17.55 μm [39]. Remarkably, this microgap was relatively larger, potentially attributed to the choice of zirconia abutments in this particular study. Study has highlighted increased wear at the implant-abutment interface of zirconia abutments compared to titanium abutments after cyclical loading [41]. While these studies reported analogous microgap measurements to our investigation, the key distinction lies in the absence of fatigue cycles in their experiments. Our study deliberately measured microgap values after loading to more authentically simulate masticatory conditions in the oral environment, potentially resulting in slightly larger microgap values than those observed without mechanical cycling.

Limitations of this study include the use of the profile cut observation method, which, while providing valuable insights, has inherent constraints in accurately measuring microgap. Future research endeavors should consider supplementing the direct observation method with indirect techniques such as bacterial culture and dye microleakage. In addition, with the accuracy range of the machines, this study was designed with only one amount of back-cutting. Accordingly, it is essential to further study to identify the optimal amount. Furthermore, the number of loading cycles and the sample size may potentially influence the outcomes. In light of the encouraging outcomes of this study, the subsequent phase could incorporate more exacting design and the utilization of more samples to evaluate the performance of the abutment. Combining multiple approaches to address the limitations of the current study allows for a more comprehensive evaluation of SLM abutments and to promote the practical implementation of the polishing technique in a clinical setting.

## Conclusion

1. The surface roughness of the SLM abutment was significantly reduced after the polishing process.
2. After polishing process, the torque loss ratio of SLM abutment before and after mechanical cycling were significantly reduced, and the microgap after mechanical cycling was significantly reduced and similar to the original abutment.
3. A notable and positive correlation existed between the surface roughness of the polished SLM abutment and both the torque loss ratio after mechanical cycling and the microgap. It implied that a decrease in surface roughness enhanced the accuracy of the SLM abutment.

## Data Availability

The complete data and materials described in the research article are freely available from the corresponding author on reasonable request.

## Abbreviations

SLM: Selective laser melting
CAD/CAM: Computer-aided design/computer-aided manufacturing
SEM: Scanning electron microscope
RTM: Recommended torque specified by the manufacturer
RTV: The removal torque value
